# Combined Effect of tDCS and GRASP for Upper Limb Rehabilitation in Stroke: A Clinical and Accelerometric Pilot Study

**DOI:** 10.3390/s25164907

**Published:** 2025-08-08

**Authors:** Erica Grange, Rachele Di Giovanni, Fabio Giuseppe Masuccio, Virginia Tipa, Luca Dileo, Alessandra Bordino, Micaela Porta, Bruno Leban, Martina Rolla, Massimiliano Pau, Claudio Marcello Solaro

**Affiliations:** 1 Scientific Research Area, Italian Multiple Sclerosis Foundation (FISM), 16149 Genova, Italy; ericagrange@gmail.com; 2 Centro di Recupero eRieducazione Funzionale “Monsignor Luigi Novarese”, Loc Trompone, 13040 Moncrivello, Italy; fgmasuccio@yahoo.com (F.G.M.); martinarolla@libero.it (M.R.); 3 Struttura Complessa di Medicina Fisica e Riabilitativa, Dipartimento Medico Generale e Riabilitativo, Azienda Sanitaria Locale Cuneo 1, 12045 Fossano, Italy; virginiatipa@gmail.com; 4 Centro “S. Maria ai Colli—Presidio Ausiliatrice”, Fondazione Don Gnocchi, 10133 Torino, Italy; dileo.ot@gmail.com; 5 A.S.P. Istituti Milanesi Martinitt e Stelline e Pio Albergo Trivulzio, via Antonio Tolomeo Trivulzio, 15, 20146 Milano, Italy; alessandra.bordino@gmail.com; 6 Department of Mechanical, Chemical and Materials Engineering, University of Cagliari, 09123 Cagliari, Italy; micaela.porta@unica.it (M.P.); bruno.leban@unica.it (B.L.); massimiliano.pau@unica.it (M.P.); 7 Neurology Unit, Galliera Hospital, 16149 Genova, Italy; csolaro@libero.it

**Keywords:** stroke, upper limb, rehabilitation, tDCS, GRASP, accelerometry

## Abstract

Upper limb (UL) impairment after stroke negatively influences stroke survivors’ quality of life (QOL). This study aims to evaluate, through clinical assessment and accelerometric measures, the efficacy of anodal Transcranial Direct Current Stimulation (a-tDCS) combined with the Graded Repetitive Arm Supplementary Program (GRASP) in post-acute stroke UL rehabilitation. Subjects were enrolled if they were aged ≥18 years and had a first stroke diagnosis, UL motor impairment and adequate trunk control. The subjects underwent combined administration of intensive a-tDCS and GRASP (15 sessions/30 min each). Before and after treatment, a subgroup of subjects was evaluated through wearable accelerometers. A total of 30 subjects were included in this study (mean age 68.34 ± 14.08 years; 19 males/11 females). Medical Research Council (MRC), Hand Grip Strength (HGS), Nine-Hole Peg Test (9HPT), Box and Block Test (BBT) and Fugl-Meyer Assessment-Upper Extremity (FMA-UE) scores significantly improved after treatment. The accelerometric-derived measurements all revealed a significant increase in the affected UL activity as indicated by the Vector Magnitude value. No side effects were reported. In conclusion, an intensive a-tDCS and GRASP application proved to be effective and safe in UL rehabilitation after stroke. The association of accelerometric monitoring might be of paramount importance for the evaluation of UL recovery.

## 1. Introduction

Stroke is the second-leading cause of death and the third leading cause of combined death and disability, as expressed by disability-adjusted life years lost in the world [1]. The effects of stroke on the upper limb (UL) functions are a common and significant source of long-term disability [2]. Symptoms such as paresis, loss of sensation, pain and spasticity can affect the hands, arms and shoulders with consequences for people’s daily lives [3]. These include a reduced capacity to carry out basic self-care tasks and activities, and to fulfill life roles, which can affect emotional and psychological wellbeing [4,5]. About 70% of stroke survivors have different degrees of UL movement dysfunction [6]. In one-third of this percentage, a severe UL impairment was present since the acute phase [7]. Furthermore, about half of stroke survivors still report an impairment of the affected UL’s functionality after 6 months from the event [8]. Therefore, the restoration of UL function to achieve independence is one of the main goals of stroke rehabilitation. Different effective rehabilitative approaches have been reported in the literature, such as mobilization, constraint-induced movement therapy, task-oriented exercises, action observation training, mirror therapy and activities of daily living (ADLs) training [9]. Recently, non-invasive brain stimulation (NIBS) techniques have been introduced for the improvement of UL motor function and performance in ADL after stroke [10]. These techniques potentially rely on the principle of neuroplasticity, inducing changes in neuronal functioning through synaptogenesis, reorganization, and network strengthening and suppression [11,12]. Among NIBS methods, Transcranial Direct Current Stimulation (tDCS) is a low-amplitude electrical stimulation where a cerebral cortex neuromodulation process occurs through the inhibition and excitability loop [13]. This rehabilitative technique has led to promising results on UL motor function recovery and performance in ADLs after acute, sub-acute and chronic stroke [10]. Two types of tDCS are provided: the anodic one (a-tDCS), which increases the cortical excitability of the affected hemisphere; and the cathodic one (c-tDCS), which is used to reduce the cortical excitability of the contralesional hemisphere. A combined bi-hemispheric a-tDCS and c-tDCS has also been explored [14]. In particular, the a-tDCS seems to have greater benefits if applied to acute and sub-acute stroke, when associated with rehabilitation [15]. However, the clinical outcome measures used to assess the efficacy of tDCS and the rehabilitation protocol delivered during the stimulation are very heterogeneous. Furthermore, there is no consensus on the best modalities of stimulation and stimulation dosage, including number of total sessions, length and frequency of the single session, and intensity of the current, or on the application of a standardized UL exercise protocol [15].

In this regard, the Graded Repetitive Arm Supplementary Program (GRASP) [16] might be a valid option to apply in combination with tDCS. Indeed, GRASP is a standardized program for UL recovery, composed of repetitive goal-oriented tasks, performed under the close observation of an Occupational Therapist (OT). The combination of a-tDCS and GRASP treatment might be effective in enhancing the recovery of UL impairment after stroke. Thus, the aim of this study is to assess the efficacy of an intensive a-tDCS application in association with a standardized UL rehabilitation protocol in sub-acute stroke survivors, and to evaluate UL performance using instrumental wearable accelerometers.

## 2. Materials and Methods

### 2.1. Subjects

This is a pilot study conducted in the Rehabilitation Center “Mons. Luigi Novarese” of Moncrivello (VC), Italy. The study participants were enrolled among those who had been admitted for intensive rehabilitation after stroke if fulfilling the following inclusion criteria: being ≥18 years old, confirmed first-ever stroke diagnosis (throughout CT or MRI neuroimaging) in sub-acute phase (>1 week and <3 months from the onset), UL motor impairment [Fugl-Meyer Assessment—Upper Extremity (FMA-UE) ≤ 58], activation of wrist and finger extension, adequate trunk control [Trunk Control Test (TCT) > 24] and ability to give informed consent and to understand the study procedure.

Subjects were excluded if having impaired cognitive function (Montreal Cognitive Assessment, MoCA < 15), severe upper limb motor impairment (FMA-UL ≤ 10), severe upper limb spasticity (Modified Ashworth Scale (MAS) ≥ 3), presence of hemispatial neglect (barrage test score), apraxia and/or aphasia (Token Test < 15). Subjects were also excluded if having pacemaker or intracranial mechanical implants; previous congenital or acquired neurological disease; severe cardiopulmonary, renal and hepatic diseases; pregnancy; drugs interfering with membrane conductivity; psychiatric disorders; alcohol or drug abuse; and/or epilepsy diagnosis.

### 2.2. Outcomes

The primary outcome of this study is the FMA-UE, a measure designed to assess the global upper limb functionality, including reflex activity, movement control, muscle strength, sensitivity and pain of the affected UL of people with post-stroke hemiplegia [17].

The secondary outcomes of this study are the Nine-Hole Peg Test (9HPT), the Box and Block Test (BBT), the Hand Grip Strength Test (HGS) and the Barthel Index (BI). Also, objective instrumental metrics of UL activity under free-living conditions, obtained using wearable accelerometers and previously demonstrated to be responsive to changes in upper extremity function of stroke survivors, were considered as secondary outcomes [18].

### 2.3. Clinical Measures

9HPT is a quantitative test of upper extremity function, evaluating fine manual dexterity [19,20]. It consists in taking nine small pegs from a container, one by one, and placing them into holes on a board, as quickly as possible, then removing each peg from the holes, and placing them back into the container. For each arm, the average time of two trials represents the final score of the test. The time taken to complete each trial was recorded with a maximum time of 300 s.

BBT is a functional outcome measure of gross manual dexterity [21]. It consists of a box (53.7 cm × 9 cm × 25.4 cm) divided by a panel into two spaces (15.2 cm high), filled with 150 blocks. The subject grasps one block at a time, moves the block over the partition and releases it into the opposite compartment as quickly as possible. The score is the number of blocks moved in 1 min, for each hand separately.

HGS quantifies the maximum isometric strength of the hand with a hydraulic dynamometer; the JAMAR^®^ Hand Dynamometer has been employed in literature [22]. The test was performed in the following position: shoulder abducted and neutrally rotated, elbow flexed at 90°, forearm in a neutral position, wrist between 0° and 30° dorsiflexion and between 0° and 15° ulnar deviation. The measure was taken in triplicate and the mean of the three trials was considered for each hand.

The Medical Research Council (MRC) score was adopted for measuring limb strength. It consists in grading strength on the basis of a patient’s ability to activate a muscle group, to move a limb segment through a range of motions and to resist the examiner’s force. The MRC score has been evaluated for shoulder abductors, elbow flexors and extensors, wrist flexors and extensors, and finger flexors and extensors [23,24].

The Modified Ashworth Scale (MAS) [25] is a clinical tool used to measure the increase in muscle tone; in this study, shoulder, elbow, wrist and finger muscles were evaluated.

The Barthel Index (BI) [26], an ordinal scale that measures the individual’s performance on 10 activities of daily living (ADLs), was administered to provide an overview of each subject’s level of autonomy [27]. The BI assesses the ability of an individual with a neuromuscular or musculoskeletal disorder to take care of himself/herself. Ten items investigate feeding, bathing, personal grooming, dressing, toilet use, bladder and bowel continence, chair transfer, walking and climbing the stairs. Every item has a score according to the modalities of execution (autonomously, with partial or complete dependence). The score ranges from 0 to 100 (0 = complete dependence; 100 = complete autonomy).

The Montreal Cognitive Assessment (MoCA) [28] is a brief cognitive screening instrument developed to, through a one-page/30-point test that takes 15 min to administer, specifically differentiate mild cognitive impairment from cognitive changes of normal aging. The total possible score is 30 points.

The Beck Depression Inventory—II edition (BDI-II) [29] is a brief, self-reported inventory developed to measure the severity of depression symptomatology; it consists of 21 groups of statements (the subject must consider how he/she has been feeling during the past two weeks).

The Trunk Control Test (TCT) [30] is a short test for motor function (in particular to measure trunk control); it examines four simple aspects of trunk movement. The patient lies supine on the bed and is asked to roll to the weak side, roll to the strong side, sit up from lying down and sit in a balanced position on the edge of the bed, with the feet off the ground for a minimum of 30 s. The score is the simple addition of the scores obtained on the four tests.

### 2.4. Upper Limb Activity Data Collection and Processing

Objective data on UL activity under free-living conditions were collected for 3 consecutive days [31] by means of two tri-axial accelerometers (Actigraph GT9X Link, ActiGraph LLC., Pensacola, FL, USA) previously employed in similar studies on individuals with stroke [32]. Participants were required to wear them on both wrists 24 h/day (in order to have available information about sleep quality), removing them only for showering or bathing. At the end of the acquisition period, the raw acceleration data (collected in 1 s epochs at 30 Hz frequency) were downloaded to a personal computer and processed by means of dedicated software (Actilife v6.13.3 ActiGraph LLC, Pensacola, FL, USA). A CSV file containing the accelerometric counts recorded for the three axes was generated and subsequently processed with a custom routine (developed under a Matlab v6.13.3 environment) to calculate the following:

- Vector Magnitude (VM) counts, which represent a composite measure of the tri-axial accelerometric counts and is calculated through the following equation:

$$VM=\sqrt{{Axis}_{1}^{2}+{Axis}_{2}^{2}+{Axis}_{3}^{2}}$$

where *Axis*_1_, *Axis*_2_ and *Axis*_3_ represent the counts calculated for each of the three orthogonal axes (vertical, longitudinal and lateral), respectively. Higher VM values indicate higher overall upper limb activity [30].

- Minutes of use: sum of the time periods (expressed in minutes) in which the VM value was higher than zero [33] calculated separately for each limb.
- Use Ratio (UR): the ratio between the minutes of use calculated for the affected and non-affected limb, regardless of performed movement intensity [33]. In the case of perfectly balanced use of the two limbs, UR assumes a value = 1. Otherwise, values lower than 1 and higher than 1 denote longer periods of use of either the non-affected or affected limb, respectively.
- Magnitude Ratio (MR): the natural logarithm of the ratio between the VM counts associated with the affected and the non-affected limb activity [33,34]. In this case, perfect symmetry (in terms of intensity of use) is indicated by a UR = 0, while negative (positive) values indicate higher intensity of activity of the non-affected (affected) limb.
- Mono Arm Use Index (MAUI): a parameter which expresses the sum of the magnitude of all independent movements of each arm (i.e., a movement of one limb when the other is inactive). MAUI, which was designed to quantify the frequency of independent movement in everyday activities, can be obtained by the following equation [35]:

$$MAUI=\frac{\sum_{\forall n/{VM}_{dom\left(n\right)=0}}{VM}_{nondom}\left(n\right)}{\sum_{\forall n/{VM}_{nondom\left(n\right)=0}}{VM}_{dom}\left(n\right)}$$

where *n* represents a certain time sample and VM the overall measure of the accelerometric counts recorded for either the affected or non-affected limb. Unity values for MAUI express perfectly symmetrical activity of the upper limbs considering their activity counts. Values lower and higher than 1 indicate unbalanced activity towards the non-affected and affected limb, respectively.

- The Bilateral Arm Use Index (BAUI) is expressed by the equation [35]

$$BAUI=\frac{\sum_{\forall n/{VM}_{dom\left(n\right)\neq 0}}{VM}_{nondom}\left(n\right)}{\sum_{\forall n/{VM}_{nondom\left(n\right)\neq 0}}{VM}_{dom}\left(n\right)}$$

with the same notation previously described. Different from MAUI, BAUI provides information on those activities which simultaneously involve both limbs. The condition for perfect symmetry as well as the interpretation of values lower and higher than 1 are the same as previously mentioned for the MAUI.

### 2.5. Treatment Protocol

The treatment protocol consisted of 15 a-tDCS sessions of 30 min combined with the application of the GRASP for UL rehabilitation.

At the baseline all the clinical and demographic characteristics of the sample were recorded, including the Bamford classification for the lesion site (PACI: partial anterior circulation infarcts; TACI: total anterior circulation infarcts; LACI: lacunar infarcts; POCI: posterior circulation infarcts) [36].

All the participants underwent two evaluations at the following timepoints:

Baseline evaluation protocol before a-tDCS treatment (T0): administration of primary and secondary outcome measures (FMA-UE, 9HPT, BBT, HGS, MRC, MAS, BI) and Bamford classification.

Final evaluation (T1): administration of primary and secondary outcome measures (FMA-UE, 9HPT, BBT, HGS, MRC, MAS, BI) after the application of the 15 sessions of a-tDCS treatment protocol.

A subgroup of participants underwent 3 consecutive days of accelerometers recording upon both T0 and T1.

### 2.6. a-tDCS Protocol

Anodal-tDCS uni-hemisphere stimulation was applied for a total of 15 sessions, specifically 5 consecutive 30 min sessions for three consecutive weeks, with a current intensity of 1.5 mA. Two saline-soaked sponge electrodes were applied: the stimulating electrode (anode, 5 × 5 cm^2^) was placed over primary motor cortex M1 (position C3/C4) of the damaged hemisphere; the return electrode (cathode, 5 × 5 cm^2^) was placed on the contralateral supraorbital area (position Fp1/Fp2) [13,37]. The current density has been maintained under safety limits reported in the literature [38]. The tDCS has been performed through the BrainSTIM (EMS, Bologna, Italy) constant current stimulator. Side effects were recorded with an ad hoc questionnaire including a Yes/No answer regarding the symptoms reported in the literature [39] after every session of tDCS.

### 2.7. Modified GRASP Protocol

In order to deliver a standardized UL rehabilitation treatment, a modified GRASP protocol was applied by an OT. It consisted of a 30 min session of exercises included in the GRASP program, performed in association with a-tDCS. GRASP [16] is an exercise program for the recovery of the hand and arm designed through challenging repetitions of practice in order to improve paretic UL performances, to encourage its use in ADL [16]. GRASP has 3 exercise stages to accommodate different levels of UL function (each level has its own participant manual), with a gradual increase in the task difficulty, based on FMA-UE score: Level 1: FMA-UE = 10–25 (Practice of gross motor skills and introduction of fine motor skills); Level 2: FMA-UE = 26–45 (Practice of gross motor skills and fine motor skills); Level 3: FMA-UE = 46–58 (Practice of gross motor skills and substantial fine motor skills). Throughout the sessions, the level of the GRASP was modulated according to each subject’s UL function improvement. In order to adapt the duration of the original GRASP to the tDCS session length, a reduced number of exercises and activities was proposed. In particular, stretching and redundant exercises were excluded, to give priority to task-oriented exercises. For both GRASP level 2 and level 3, two distinct exercise protocols were developed and delivered on alternate days to ensure varied motor stimulation and to promote engagement in diverse activities. In addition, the authors adapted the number of repetitions or series of the exercises to make the levels ever more challenging with an increase of 5 repetitions per series or an increase of an entire series per exercise. During each session, subjects were asked to rate the effort degree of each exercise on an NRS ranging from 0 to 10, and side effects were recorded.

The modified GRASP program level 1, 2 and 3 protocols are available in the Supplementary Materials.

### 2.8. Statistical Analysis

Due to the non-normal distribution of the clinical parameters (primary and secondary outcome measures), non-parametric tests were utilized. The Mann–Whitney U-test was used to investigate sex-related differences and to compare those who underwent accelerometry and those who did not. The presence of differences in strength and parameters of UL function over time was investigated through the Wilcoxon test. The level of significance was set at *p* = 0.05 and the appropriate effect size was calculated through the zeta (Z) coefficient.

Instead, the existence of possible changes in UL activity and symmetry of use parameters introduced by the treatment was investigated using either one- or two-way analysis of variance for repeated measures (RM-ANOVA). In particular, in the case of VM counts, time (pre- or post-treatment) and limb (affected/non-affected) were set as independent variables, while VM count values served as the dependent variable. Instead, since symmetry parameters consider both limbs, the only independent variable was time and the dependent variables were the symmetry parameters (i.e., UR, MR, MAUI and BAUI). The level of significance was set at *p* = 0.05 and the effect sizes were assessed using the eta-squared (η^2^) coefficient.

We also explored the existence of a possible relationship between the overall upper limb activity (described using VM counts); the performance in BBT, 9-HPT and HGS tests; and the FMA-UE score (in this latter analysis only data of the affected limb were considered) using Spearman’s rank correlation coefficient rho. Even in this case the level of significance was set at *p* = 0.05. All analyses were performed using the IBM SPSS Statistics v.20 software (IBM, Armonk, NY, USA).

## 3. Results

A total of 30 participants (19 males and 11 females; mean age 68.34 (14.08) years) who were admitted for intensive rehabilitation following their first stroke were enrolled in this study, as they met all the inclusion criteria. Among these individuals, 2 had experienced a hemorrhagic stroke, while the remaining 28 had suffered an ischemic stroke; 14 had right-sided hemiparesis and 16 left-sided hemiparesis (Table 1).

A subgroup of 16 subjects (10 males and 6 females; mean age 66.18 ± 13.17 years) underwent accelerometric measurements through wearable accelerometry. There were no differences upon T0 and T1 in terms of FMA-UE, 9HPT, BBT, HGS, MRC and MAS or between males and females, or between those who underwent accelerometric measurements and those who did not.

Table 2 presents the clinical assessment scores recorded upon T0 and T1, focusing on measures such as FMA-UE, 9HPT, BBT, HGS, MRC, MAS and BI.

Upon T1, the MRC scores for muscle groups including the triceps, biceps, shoulder abductors, wrist flexors and extensors, and finger flexors and extensors showed a significant improvement. This indicates an enhancement in the overall strength of the affected UL.

Regarding UL functionality, the FMA-UE, HGS and the BBT upon T1 augmented significantly, suggesting an enhancement in the functional performance of the affected arm and hand. In addition, the 9HPT scores decreased significantly, thus with a faster completion time, further indicating improved dexterity and motor control of the affected UL.

Upon T1 BI increased significantly, highlighting a higher degree of independence after treatment compared to the T0 levels.

On the other hand, the MAS score, assessing muscle hypertonia, did not show significant changes over time, indicating that the treatment did not exacerbate hypertonia.

The registration of side effects revealed the absence of side effects both during and after the tDCS sessions.

Regarding the GRASP levels, 7 subjects completed level 1, 9 subjects completed level 2, and 12 subjects completed level 3. Only two subjects changed the GRASP level over time (one from level 1 to level 2; one from level 2 to level 3).

### Accelerometry-Based Metrics

The results of the accelerometric-derived metrics are summarized in Table 3.

It is noteworthy that all participants demonstrated excellent compliance with the experimental protocol, as the average wear time was 87.7%. Even in the worst case, it was still possible to process 15 valid hours per day, a value consistent with the recommended minimum time necessary to obtain reliable data on physical activity in individuals with stroke using accelerometers [31].

**Figure 1:**
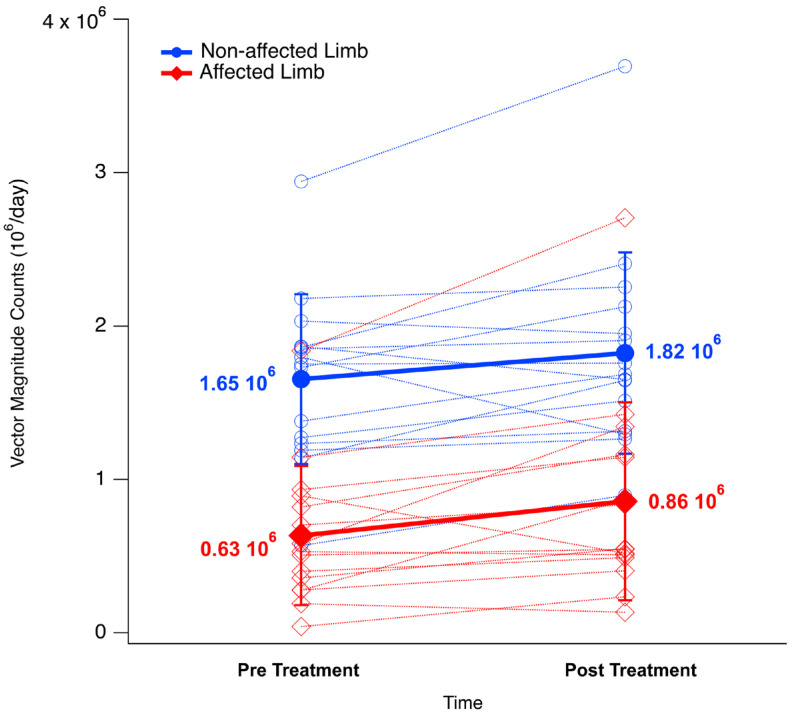
Changes in participants (dashed lines) and whole group (thick lines) mean values of the accelerometric-derived measure of upper limb mobility (Vector Magnitude) before and after the treatment for the affected (red) and non-affected (blue) limb.

RM-ANOVA detected a significant main effect of time [F (1,14) = 6.14, *p* = 0.027, η^2^ = 0.02] and limb [F (1,14) = 75.40, *p* < 0.001, η^2^ = 0.43] on overall UL activity as represented by VM counts (Figure 1). However, the post hoc analysis revealed that the effect of the treatment significantly increased only the mobility of the affected limb (*p* = 0.015), although for the non-affected limb, significance was almost achieved (*p* = 0.055). As expected, significant differences across the two limbs were found before and after the treatment (*p* < 0.001 in both cases). In contrast, no significant changes associated with the treatment were observed as regards the symmetry of use parameters (UR [F (1,22) = 1.26, *p* = 0.304, η^2^ = 0.01], MR [F (1,22) = 1.83, *p* = 0.225, η^2^ = 0.005], MAUI [F (1,22) = 0.603, *p* = 0.467, η^2^ = 0.005] and BAUI [F (1,22) = 4.14, *p* = 0.088, η^2^ = 0.014]).

The results of the correlation analysis (Table 4) indicate the existence of significantly large positive correlations between the raw accelerometric counts (VM) and the results of the BBT (rho = 0.777, *p* < 0.001) and with those of the HGS test (rho = 0.773, *p* < 0.001), while a large negative correlation was found with the performance in the 9-HPT (rho = −0.857, *p* < 0.001). In contrast, no significant correlation was found between the VM and FMA-UE score.

## 4. Discussion

The hypothesis that the association between motor training and a-tDCS might increase motor function after stroke has already been reported [40]. However, to the best of our knowledge [10,41], this is the first pilot study introducing a-tDCS stimulation in combination with a standardized and validated rehabilitative protocol for UL recovery after stroke, GRASP. The two treatments were administered simultaneously, in order to better stimulate neuroplasticity with a-tDCS and motor exercise [10]. Regarding the stimulation method, we have chosen a-tDCS, an excitatory protocol, due to the demonstrated higher efficacy in respect to c-tDCS on UL recovery after stroke [10]. When considering the use of a standardized training program for UL rehabilitation, it has been demonstrated that GRASP administration can ameliorate UL function after stroke [16]. Indeed, this exercise set fits well with an inpatient setting, as it needs to be repeated continuously. Furthermore, GRASP is conducted by the patient under the guidance of the OT, providing easy-to-perform actively targeted exercises, which are largely recognized as being the most effective on neuroplasticity [16,41].

The results of our study suggest that UL functionality improved after the application of the a-tDCS/GRASP treatment. In this regard, although in a small sample, the accelerometric measures confirmed the efficacy of the used techniques. When analyzing the findings, upon T1, a significant decrease of 9HPT and an increase of FMA-UE, BBT and HGS were documented. In addition, a significant increase in UL utilization was detected through wearable accelerometers. The reliability and validity of FMA-UE, 9HPT, BBT and HGS for the UL evaluation are well recognized, and it is also well documented that these outcome measures appropriately reflect arm and hand function [3]. Furthermore, the accelerometric-derived metrics provided further insights regarding the effectiveness of the treatment under real-life conditions. In fact, we observed a significant increase in the activity of the affected limb consequent to the intervention, as indicated by the VM value (+36%), a parameter usually considered a reliable proxy for the overall UL mobility, as it encompasses the arm movement associated with ADLs and gait. Although in this case the statistical significance was not achieved (and thus the results should be considered cautiously), the analysis of the parameters more directly related to the symmetry of UL use suggests that the stimulation originated a trend towards a more balanced use of the two limbs, by shifting activity from the non-affected to the affected limb for both uni- and bimanual activities. To the best of our knowledge, although no previous studies investigated the effect of tDCS in terms of real-life mobility using accelerometers, it is noticeable that recent reviews reported that this kind of stimulation is beneficial to some extent in terms of clinical outcomes [42] and ADLs [41]. Thus, it is possible to hypothesize that the improvements observed in the accelerometric-based parameters are associated with a general increase in mobility and a superior engagement in ADLs, which benefits from a certain degree of recovery in terms of use of the non-affected limb.

It is also worth noting that the VM showed a strong correlation with clinical measures of gross and fine dexterity and with handgrip strength. This link, which was previously also observed in the case of other neurologic diseases [43], reinforces the idea that longitudinal instrumental monitoring of UL activity can provide useful information about the effect of a rehabilitative treatment. This might effectively integrate the clinical information (which is sporadic in nature) with data concerning not only overall mobility but also other movement features of interest. In contrast, it was only partly surprising to observe that accelerometric data were not correlated with the FMA-UE score.

Indeed, it is known that the FMA-UE focuses on the impairment level of motor control, not on the real-world functional use of the limb. As such, FMA-UE is not able to fully capture the way people with stroke use their arm in daily activities (e.g., dressing, cooking), a fact limiting its ecological validity. Also, FMA-UE is administered under controlled clinical conditions (often at maximum effort) with verbal encouragement and full focus. In contrast, accelerometers measure what the individual actually does in daily life under less ideal conditions (e.g., fatigue, distraction or lack of motivation). The resultant “capacity–performance” gap well represents the fact that functional ability assessed through clinical scales does not always translate to actual real-world usage.

Undoubtedly, another noticeable finding is represented by the lack of modification of MAS over time after a-tDCS and GRASP application. When analyzing previous reports [44,45,46], data regarding post-stroke spasticity treatment with tDCS are inconsistent. In particular, a moderate-to-low quality of evidence was found for the absence of effects of tDCS in improving spasticity after stroke, additionally without any long-term follow-up data [47]. However, the low degree of spasticity detected in our sample might have had an influence on this result.

Due to the nature of this study, no control group was enrolled and this represents a formal limitation. However, stating the tolerability of this prolonged combined a-tDCS and GRASP treatment was a precise need in conducting this pilot study. In particular, in the context of an inpatient setting, this might not be tolerated by the subjects recruited due to the effects of an intensive rehabilitation program. However, we did not detect any side effects and all the participants tolerated the whole treatment well, laying the foundations for the implementation of randomized clinical trials. Moreover, the participants did not report any itching, dizziness, tingling, discomfort, headache or burning sensations. This is not in line with previous studies demonstrating a prevalence of these side effects ranging from about 10 to 39% [39], but the small sample of participants recruited might be the principal reason for this disagreement. Formally, the sample numerosity represents another limitation of this study. However, it has to be considered that this is a highly selective population of stroke survivors who had been admitted to rehabilitation after their first-ever stroke, with a wide variety of clinical features. In particular, in order to perform GRASP correctly, participants should have experienced activation of wrist and finger extensors. In addition, we recruited subjects having an adequate trunk control ability (TCT > 24) to provide more precise measures of UL function. Actually, the trunk control is necessary for balance and posture while the individual moves the UL. Thus, it is a fundamental requirement not only for UL function but also for an appropriate UL evaluation [48]. Another necessary condition for recruitment was the possibility of giving informed consent and understanding the study procedure. Indeed, cognitive impairment and aphasia very often accompany stroke; thus, we selected the participants in order to avoid possible confounding factors.

## 5. Conclusions

Among new techniques available, NIBS, and in particular tDCS, might be of fundamental importance for the recovery of impaired UL after stroke, even combined with monitoring of accelerometric measures.

In the present study, a-tDCS and GRASP proved to be safe and effective for the recovery of UL impairment in sub-acute stroke survivors. In particular, both FMA-UE and VM significantly rose in association with a consensual increase of BI, highlighting a potential role of the combination of a-tDCS and GRASP in the rehabilitation of stroke-related UL dysfunction.

The combination of a-tDCS and GRASP might also be applied in an outpatient setting or at home. In this regard, long-term RCT should be conducted, also introducing sham a-tDCS. This might allow us to better understand the modifications of UL function during the application of a-tDCS and a standardized rehabilitative protocol for UL recovery, such as GRASP.

## Figures and Tables

**Table 1:** Clinical and demographic characteristics of the sample (expressed as mean and SD or relative distribution of frequencies, as appropriate).

Number of subjects	30
Males/Females	18 (60%)/12 (40%)
Schooling (years)	9.10 (4.35)
Stroke type	
Ischemic/Hemorrhagic	28 (93.30%)/2 (6.70%)
Body side affected	
Right/Left	15 (50%)/15 (50%)
Handedness	
Right/Left	28 (93.30%)/2 (6.70%)
Bamford classification	
PACI	5 (17.90%)
TACI	7 (25%)
LACI	5 (17.90%)
POCI	11 (39.20%)
Autonomous walking before stroke	30 (100%)
Functional independence before stroke	30 (100%)
BDI-II	12 (8.23)
MoCA (adjusted score)	20.38 (3.84)
TCT	65.4 (29.07)

PACI: partial anterior circulation infarcts; TACI: total anterior circulation infarcts; LACI: lacunar infarcts; POCI: posterior circulation infarcts; BDI-II: Beck Depression Inventory—II edition; MoCA: Montreal Cognitive Assessment; TCT: Trunk Control Test.

**Table 2:** Comparison between pre- and post-treatment clinical parameters of the affected upper limb in the entire sample (AUL). Values are expressed as mean (SD).

Parameter (AUL)	T0	T1	Z **	p *
FMA-UE	38.65 (15.37)	50.72 (10.98)	−4.532	0.0004 †
9HPT (s)	185.44 (122.61)	120.66 (114.76)	−4.107	0.0001 †
BBT (blocks)	19.55 (16.9)	30.13 (17.97)	−4.523	0.0004 †
HGS (kg)	6.79 (9.81)	9.53 (9.62)	−3.989	0.0001 †
MRC-Triceps	3.27 (1.22)	4.05 (0.90)	−3.636	0.0006 †
MRC-Biceps	3.31 (1.07)	4 (1)	−3.947	0.0001 †
MRC-Shoulder (Abductors)	3.03 (1.35)	3.9 (0.98)	−3.686	0.0002 †
MRC-Wrist flexors	3.10 (1.37)	3.9 (1.11)	−3.750	0.0004 †
MRC-Wrist Extensors	3.07 (1.46)	3.72 (1.33)	−3.727	0.0001 †
MRC-Finger Flexors	3.07 (1.46)	3.9 (1.14)	−3.714	0.0002 †
MRC-Finger Extensors	2.90 (1.54)	3.55 (1.27)	−3.256	0.001 †
MAS-Triceps	0.13 (0.57)	0.10 (0.55)	−1.000	0.317
MAS-Biceps	0.28 (0.54)	0.23 (0.50)	−1.134	0.257
MAS-Shoulder (Adductors)	0.13 (0.41)	0.13 (0.43)	0	1.000
MAS-Wrist Flexors	0.18 (0.48)	0.17 (0.46)	−0.276	0.783
MAS-Wrist Extensors	0.10 (0.55)	0.07 (0.36)	−1.000	0.317
MAS-Finger Flexors	0.18 (0.62)	0.20 (0.55)	−0.272	0.785
MAS-Finger Extensors	0.05 (0.27)	0.07 (0.36)	−1.000	0.317
BI	44.82 (19.01)	76.72 (21.01)	−4.794	0.0001 †

FMA-UE: Fugl-Meyer Assessment—Upper Extremity; 9HPT: 9-Hole Peg Test; HGS: Hand Grip Strength test; BBT: Box and Block Test; BI: Barthel Index; MRC: Medical Research Council; MAS: Modified Ashworth Scale. † significant difference vs. pre-treatment. * *p* < 0.05 (Wilcoxon test). ** Z coefficient (Wilcoxon test).

**Table 3:** Comparison between upper limb activity parameters pre and post treatment. Values refer to the whole 3-day period and are expressed as mean (SD).

Parameter		T0	T1
Vector Magnitude (10^6^ counts/day)	Affected Limb	0.63 (0.45)	0.86 (0.64) †
	Non-affected Limb	1.65 (0.55)	1.82 (0.65)
Use Ratio *		0.54 (0.26)	0.64 (0.24)
Magnitude Ratio **		−1.19 (0.69)	−0.95 (0.58)
Mono Arm Use Index (MAUI) *		0.23 (0.22)	0.30 (0.29)
Bilateral Arm Use Index (BAUI) *		0.65 (0.12)	0.68 (0.12)

The symbol † indicates significant difference vs. pre-treatment (*p* < 0.05). * Lower values indicate superior activity on the non-affected limb. Use Ratio = 1 indicates perfect symmetry. ** Negative (positive) values indicate higher activity intensity on the non-affected (affected) limb. Higher negative (positive) values correspond to higher imbalance towards the non-affected (affected) limb.

**Table 4:** Spearman’s rho coefficient of correlation between accelerometric data (Vector Magnitude) and clinical tests for gross and fine dexterity and handgrip strength.

Vector Magnitude vs.	Spearman’s rho
FMA-UE	NS
9HPT	−0.857 *
BBT	0.777 *
HGS	0.773 *

The symbol * denotes statistical significance (*p* < 0.001); NS: non-significant. FMA-UE: Fugl-Meyer Assessment—Upper Extremity; 9HPT: 9-Hole Peg Test; HGS: Hand Grip Strength test; BBT: Box and Block Test.

## Data Availability

Anonymized data regarding this paper are available on reasonable request to the corresponding author by any qualified investigator.

## Supplementary Materials

The following supporting information can be downloaded at https://www.mdpi.com/article/10.3390/s25164907/s1: Supplementary Materials contain the table of the GRASP Progress Tracker of the activities proposed to the enrolled subjects.

## Abbreviations

The following abbreviations are used in this manuscript:

9HPT	Nine-Hole Peg Test
η^2^	Eta-squared
ADL	Activities of daily living
a-tDCS	Anodal Transcranial Current Stimulation
AUL	Affected upper limb
BAUI	Bilateral Arm Use Index
BBT	Box and Block test
BDI-II	Beck Depression Inventory—II edition
BI	Barthel Index
c-tDCS	Cathodic Transcranial Current Stimulation
FMA-UE	Fugl-Meyer Assessment—Upper Extremity
GRASP	Graded Repetitive Arm Supplementary Program
HGS	Hand Grip Strength
LACI	Lacunar infarcts
MAS	Modified Ashworth Scale
MAUI	Mono Arm Use Index
MoCA	Montreal Cognitive Assessment
MR	Magnitude Ratio (MR)
MRC	Medical Research Council
NIBS	Non-invasive brain stimulation
OT	Occupational therapist
PACI	Partial anterior circulation infarcts
POCI	Posterior circulation infarcts
QOL	Quality of life
RM-ANOVA	Analysis of variance for repeated measures
SD	Standard Deviation
TACI	Total anterior circulation infarcts
TCT	Trunk Control Test
tDCS	Transcranial Direct Current Stimulation
UL	Upper limb
UR	Use Ratio
VM	Vector Magnitude
