# Supplementary material for: Combined Effect of tDCS and GRASP for Upper Limb Rehabilitation in Stroke: A Clinical and Accelerometric Pilot Study
Source: Sensors (Basel). 2025 Aug 8;25(16):4907. doi: 10.3390/s25164907 (PMC12389919; doi:10.3390/s25164907)
Supplement: Supplementary file 1 [file sensors-25-04907-s001.zip › sensors-3721846-supplementary.pdf]

Supplementary materials

Modified version of the “Grasp Progress Tracker” with the activities performed during the treatment.

GRASP PROGRESS TRACKER

Livel #1

	ITEMS	#SETS	#REPETITIONS	EASY? HARD? (0-10)	NOTE/TIPS
1	Shoulder shrugs	1	5		
2	Push-ups	1	10		
3	One arm push-ups	1	10		
4	Shoulder exercise: arm to front	2/3	5		
5	Shoulder exercise: arm to side	2/3	5		
6	Elbow exercise	2/3	5		
7	Wrist exercise – part 1 and 2	2/3	5		
8	Grip power	2/3	5		
		2	8		
9	Squeeze the ball	2/3	5		
		2	8		
10	Finger power	1	5		
11	Waiter - cup	1	2		
12	Start the ball rolling –no partner	1	20		
13	Wash cloth twist	2/3	5		
		2	8		

Level #2_A

	ITEMS	#SETS	#REPETITIONS	EASY? HARD? (0-10)	NOTE/TIPS
1	Shoulder shrug	1	5		
2	One arm push-ups	3/2/3	5/8/8		
3	Shoulder exercise: arm to front	3/2/3	5/8/8		
4	Elbow exercise	3/2/3	5/8/8		
5	Drying off	1	10/20/30		
6	Wrist exercise – part 1 and 2	3/2/3	5/8/8		
7	Finger power	1	5		
8	Buttons	1	3		
9	Lego	1	10/20/30 lego		
10	Cutting	1	5/10		
11	Start the ball rolling: no partner	1	20		
12	Advanced waiter	1	20		
13	Block towers	1	10/20/30 blocks		

Level #2_B

	ITEMS	#SETS	#REPETITIONS	EASY? HARD? (0-10)	NOTE/TIPS
1	Push-ups	3/2/3	5/8/8		
2	Chair ups	3/2/3	5/8/8		
3	Shoulder exercise: arm to side	3/2/3	5/8/8		
4	Waiter - cup	2/2/3	2/5/5		
5	Elbow exercise	2/3**	5		
6	Wrist exercise – part 1 and 2	2/3**	5		
7	Grip power	2/3	5		
		2	8		
8	Finger twist	3/2/3	5/8/8		
9	Finger strength	1	5		
10	Start the ball rolling - advanced	1	20		
11	Wash cloth twist	2/3	5*		
		2	8*		

Level #3_A

	ITEMS	#SETS	#REPETITIONS	EASY? HARD? (0-10)	NOTE/TIPS
1	Push-ups	2/2/3	8/10/10		
2	Chairs-ups	2/2/3	8/10/10		
3	Shoulder exercise: arm to side	2/2/3	8/10/10		
4	Waiter	3+2(quick)	5		
5	Wrist exercise – part 1	2/2/3	8/10/10		
6	Grip power	2/2/3	8/10/10		
7	Finger twist	2/2/3	8/10/10		
8	Hanging up the clothes	1	10/20/30 pegs		
9	Paper clips chain	1	10/20/30 clips		
10	Jars	1	10/20/30 opening and closings		
11	Cutting	1	5/10		
12	Start the ball rolling – no partner	1	20		
13	Drop and catch	1	20 times weaker hand/20 times stronger		
14	Block towers	1	10/20/30* blocks		

Level #3_B

	ITEMS	#SETS	#REPETITIONS	EASY? HARD? (0-10)	NOTE/TIPS
1	One arm push-ups	2/2/3	8/10/10		
2	Shoulder exercise: arm to front	2/2/3	8/10/10		
3	Elbow exercise	2/2/3	8/10/10		
4	Wrist exercise – part 2	2/2/3	8/10/10		
5	Finger power	5	1		
6	Advanced waiter	3	5		
7	pouring	1	20		
8	Drop and catch	1	20 times weaker hand/20 times stronger		
9	Bouncing the ball with a partner	1	20		
10	Buttons	1	5 buttons		
11	Lego	1	10/20/30 lego		
12	Pick up sticks	1	10/20/30		
13	Poker chips	1	10/20/30		
14	Drying off	1	10/20/30		
